# Solitary Fibrous Tumor of the Kidney: A Case Report and Literature Review

**DOI:** 10.7759/cureus.490

**Published:** 2016-02-11

**Authors:** Dzmitry Fursevich, Edward Derrick, Matthew C O'Dell, Swetha Vuyyuru, Jeremy Burt

**Affiliations:** 1 Diagnostic Radiology, Florida Hospital-Orlando; 2 American University of Antigua, Florida Hospital-Orlando

**Keywords:** solitary, fibrous, tumor, neoplasm, kidney

## Abstract

Solitary fibrous tumors are neoplasms of mesenchymal origin that may occur virtually in any body part, most commonly arising from the pleura. Solitary fibrous tumor of the kidney is exceptionally rare, and limited clinical knowledge regarding its behavior makes prognosis of the neoplasm difficult. We report a case of solitary fibrous tumor of the left kidney and describe its clinical, imaging, and pathological features.

## Introduction

Solitary fibrous tumor of the kidney is a rare entity with less than 50 cases described in literature [1-3]. Its rarity and nonspecific imaging features make diagnosis of the neoplasm difficult. We report a case of solitary fibrous tumor of the left kidney exhibiting both benign and malignant characteristics, describe its imaging and pathologic features, and perform a literature review. Informed consent from the patient was not required for this study.

## Case presentation

**Figure 1:**
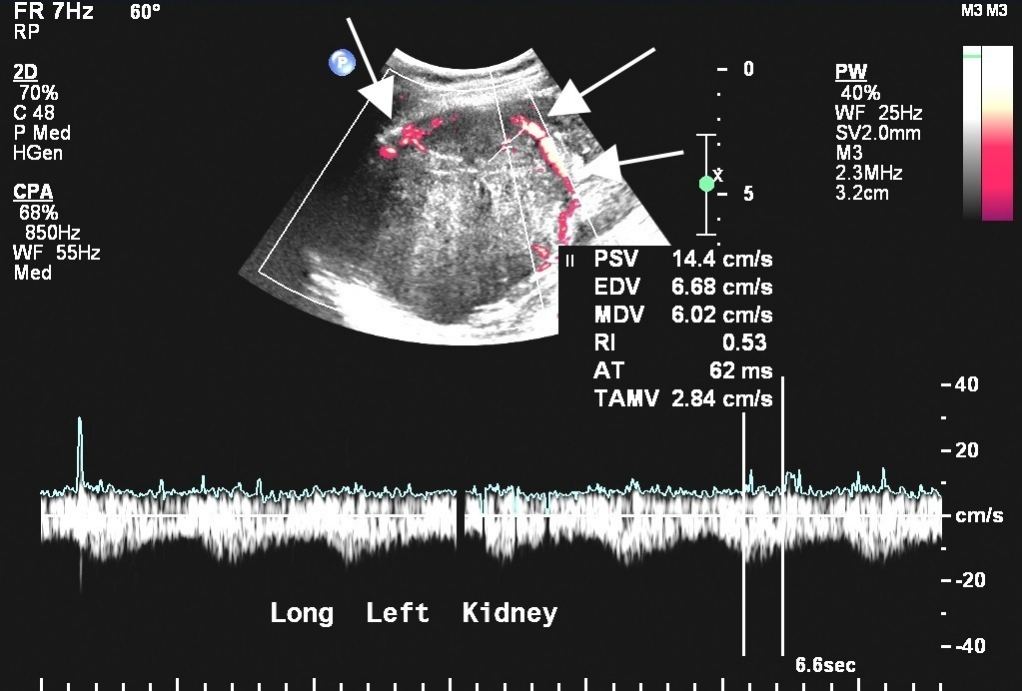
Ultrasound appearance of the SFT. A Doppler ultrasound image of the left kidney demonstrates a heterogeneous, predominantly hyperechoic left renal mass with peripheral color flow (white arrows).

A 66-year-old female with no significant past medical history presented to her primary care physician with new onset hematuria. She had palpated a mass in her abdomen eight months prior to presentation, but did not seek medical care at that time. Physical examination revealed a soft, nontender abdomen with a palpable mass in the deep left upper quadrant. Urinalysis confirmed hematuria but was otherwise unremarkable. An abdominal ultrasound revealed a heterogeneous hypoechoic left renal mass with vascular flow and areas of posterior acoustic shadowing suggestive of calcifications within the mass (Figure 1).

**Figure 2:**
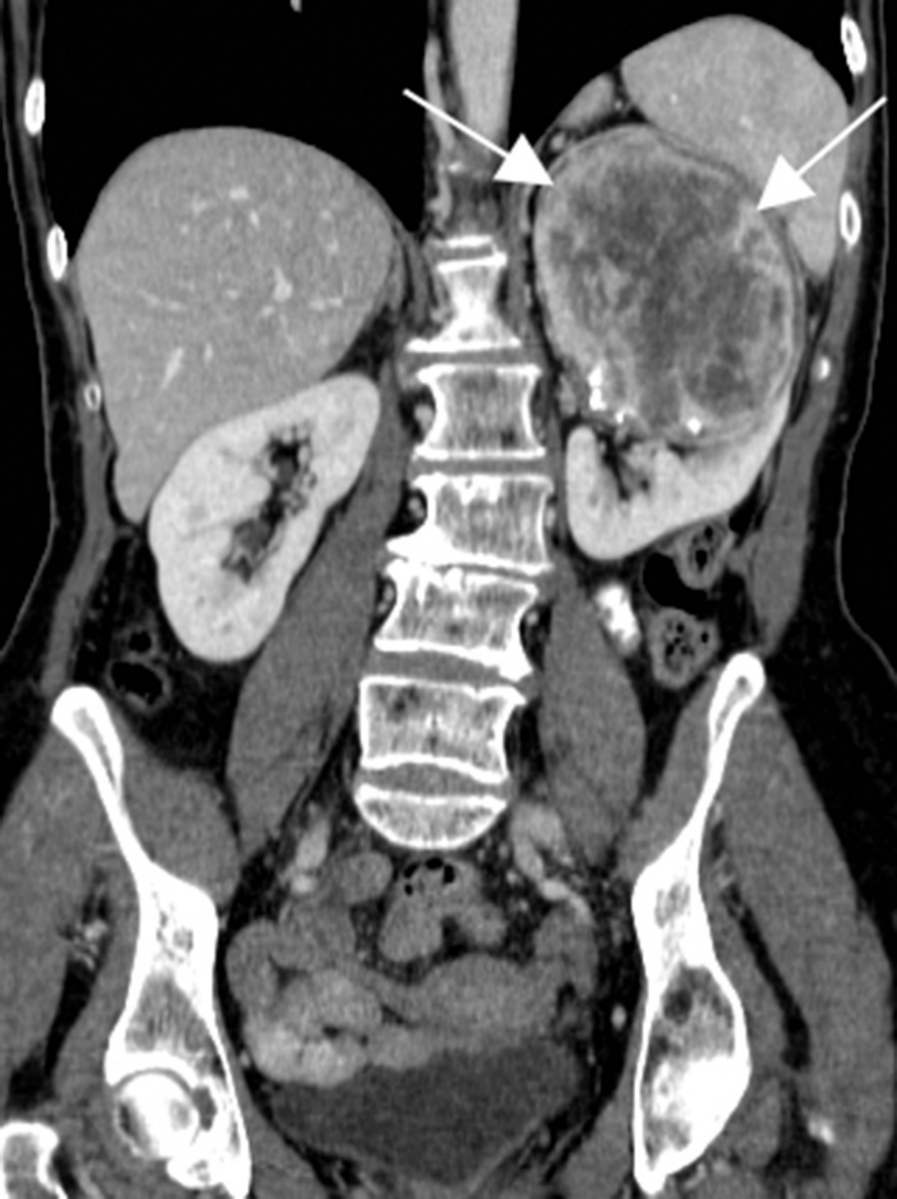
CT appearance of the SFT. A coronal contrast-enhanced CT image demonstrates a heterogeneous peripherally enhancing mass in the upper pole of the left kidney (white arrows).

Further evaluation with a contrast-enhanced abdominal CT (computed tomography) confirmed mass localization to the upper pole of the left kidney (Figure 2).

The mass was well-circumscribed and contained areas of enhancement as well as central necrosis. Calcifications were noted in the periphery of the mass without evidence of active hemorrhage. There was no CT evidence of vascular invasion or metastases. The right kidney had normal imaging appearance.

Based on the clinical and imaging features, differential diagnosis of renal cell carcinoma versus oncocytoma was considered. To exclude the rare possibilty of uroepithelial involvement and for treatment planning, the patient underwent cystourethroscopy and ureteroscopy which were negative for urothelial lesions. Left renal pelvis could not be evaluated with ureteroscopy due to the narrowing of the proximal ureter by the mass near the ureteropelvic junction. The patient then underwent a left nephrectomy.

**Figure 3:**
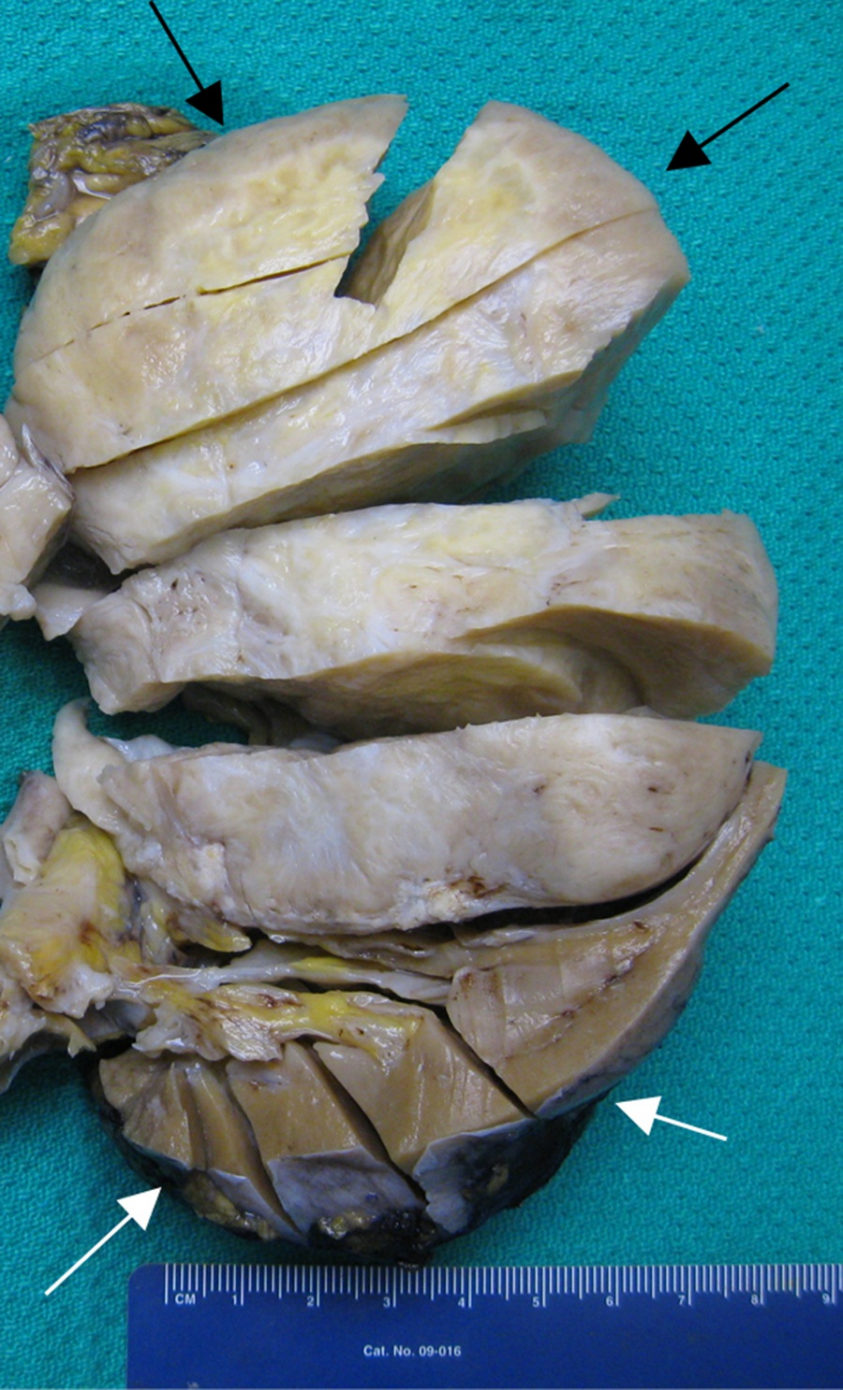
Gross specimen of the SFT. Cut section of the resected left kidney shows a rubbery pink surface of the mass in the upper pole (black arrows) with central bands of fibrosis and interspersed necrosis. Normal renal parenchyma is seen along the lower pole (white arrows).

Gross pathologic appearance revealed a solid, encapsulated mass measuring 9.3 x 7.9 x 9.4 cm (Figure 3).

Along the inferior margin of the mass were coarse calcifications, and the entire mass appeared to be surrounded by a thin rim of renal cortex. The mass caused severe compression of the calyces, renal pelvis, and renal parenchyma but showed no involvement of the renal vein. On cut section, the mass demonstrated a rubbery pink, moderately fleshy whorled surface with central tan-white fibrosis and patchy yellow necrosis.

**Figure 4:**
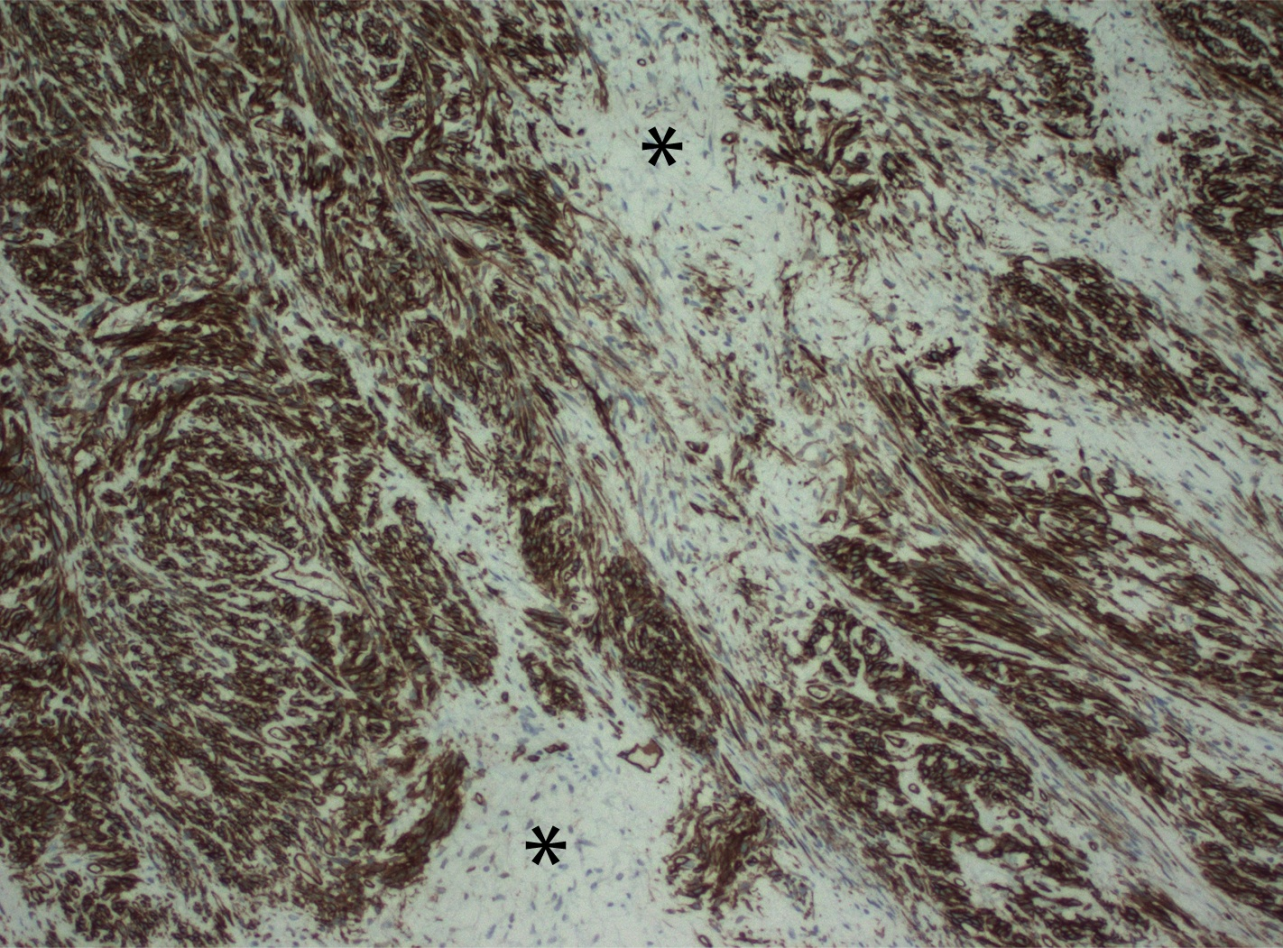
Histopathological appearance of the SFT. Histopathological CD34 slide from a solitary fibrous tumor demonstrates hyper- and hypocellular areas with bland spindle cells forming fascicles that are separated by collagen (asterisks). CD34 staining (brown) shows diffuse immunoreactivity.

Histological examination revealed a neoplasm composed of spindle cells arranged in intersecting fascicles, some of which were in a focal storiform pattern. Areas of thick collagen deposition were present and there were numerous branching, hemangiopericytoma-like blood vessels present throughout the tumor. The tumor was hypercellular with areas of necrosis, but no cellular atypia was present. Immunostaining revealed tumor cells to be diffusely positive for CD34 and BCL2 and focally positive for CD99 (Figure 4). 

The tumor cells stained negative for smooth muscle actin, caldesmon, S-100, AE1/3, HMB45, and CD117. The results of immunostaining were compatible with solitary fibrous tumor of the left kidney.

A follow up magnetic resonance imaging (MRI) of the abdomen showed no evidence of tumor recurrence or metastatic disease two years after nephrectomy.

## Discussion

Solitary fibrous tumor (SFT) of the kidney is a rare neoplasm of mesenchymal origin that has been described in patients ranging from 4 to 85 years of age [4-5]. No clear gender predilection has been established [6]. Presenting symptoms of renal SFTs include hematuria, flank pain, and enlarging abdominal mass, although these neoplasms are often discovered incidentally on imaging studies [7]. Hypoglycemia and paraneoplastic syndromes can occur in extrapleural SFTs [1], however, no cases described these symptoms in renal SFTs to date.

Imaging findings of renal SFTs are attributed to variable cellular composition, dense collagen content, and hemangiopericytomatous vascular pattern [8]. Some of the tumors may contain fat [3]. CT characteristics of renal SFTs are often nonspecific and include variable heterogeneous enhancement, calcifications, and areas of central necrosis. An MRI can help in the detection of fibrosis and dense collagen content as low signal intensity on T2-weighted images; however, these features overlap with papillary renal cell carcinoma. A nonspecific spoke wheel pattern of enhancement, similar to that of renal oncocytoma, has also been described in renal SFT [9]. Therefore, SFT may be considered in the differential diagnosis of renal tumors seen on MRI that are well-circumscribed and low in T2 signal or have a spoke wheel pattern of enhancement.

Pathologically, SFTs are fibroblastic-type mesenchymal neoplasms that can be classified as fibrous or celluar based on the predominant histopathologic findings. Although certain subtypes of SFTs have been historically characterized as hemangiopericytomas, the most recent World Health Organization classification of soft tissue tumors now classifies most hemangiopericytomas as a subtype of SFTs [10]. The fibrous variant of SFT typically has a strong CD34 reactivity at immunocytochemical analysis, whereas the cellular variant has weak CD34 reactivity and displays a hemangiopericytomatous vascular pattern [10-11].

Although some SFTs have a distinct histopathologic appearance, no strict correlation has been found between tumor morphology and its behavior [12]. Even though extrapleural SFTs have a 10-15% chance of aggressive growth and malignancy [1], only one case described pulmonary metastases in a renal SFT that invaded beyond the renal capsule and underwent sarcomatous degeneration [13]. No clear evidence-based guidelines for treatment of these tumors have been described, and the roles of preoperative biopsy and nephron-sparing surgery in lieu of nephrectomy are unclear. Most authors agree that because of their malignant potential, renal SFTs should be surgically removed. Local recurrence of renal SFTs after nephrectomy has never been documented [14-15], and there was no evidence of tumor recurrence in our case two years after surgery. However, one case described a metachronous SFT on the contralateral side eight years after left nephrectomy, suggesting that postoperative imaging surveillance should play a role in renal SFT management [16].

## Conclusions

Renal SFTs are rare mesenchymal neoplasms with potential for aggressive behavior. In most cases, the imaging characteristics are nonspecific, and the role of preoperative biopsy has not been definitely established. Although no clear evidence-based treatment guidelines exist, it is widely accepted that renal SFTs need to be removed surgically to avoid the potential of malignant degeneration and metastases. The length of post-resection follow-up imaging has not been established, and further studies are needed to determine the optimal management of these neoplasms.
